# Sustainable Chloride Removal from Conservation Electrolytes Using Alkali-Activated Carbon Nanofiber-Supported BiOCl in Capacitive Deionization

**DOI:** 10.3390/nano16150907

**Published:** 2026-07-24

**Authors:** Aoze Li, Fanghui Pan, Liping Sun, Mengying Xu, Ran Zhang, Fei Yu, Jie Ma

**Affiliations:** 1 Water Resources and Water Environment Engineering Technology Center, Xinjiang Key Laboratory of Synthesis and Application of Carbon Nanomaterials, School of Civil Engineering, Kashi University, Kashi 844000, China; aozeli@umass.edu (A.L.); m230501321@st.shou.edu.cn (L.S.); jma@tongji.edu.cn (J.M.); 2 Stockbridge School of Agriculture, University of Massachusetts, Amherst, MA 01003, USA; 3 Research Center for Environmental Functional Materials, State Key Laboratory of Water Pollution Control and Green Resource Recycling, College of Environmental Science and Engineering, Tongji University, Shanghai 200092, China; 4 National Museum of China, Beijing 100006, China; xumengying@chnmuseum.cn (M.X.); zhangran@chnmuseum.cn (R.Z.); 5 Key Scientific Research Base of Metal Conservation (National Museum of China), National Cultural Heritage Administration, Beijing 100006, China; 6 College of Oceanography and Ecological Science, Shanghai Ocean University, No 999, Huchenghuan Road, Shanghai 201306, China; 7 Shanghai Institute of Pollution Control and Ecological Security, Shanghai 200092, China

**Keywords:** capacitive deionization, chloride removal, bismuth oxychloride, carbon nanofibers, bronze conservation

## Abstract

Chloride-induced corrosion is a major threat to excavated bronze artifacts, yet conventional alkaline desalination requires repeated solution replacement and generates secondary chemical waste. Herein, a series of BiOCl-loaded carbon nanofiber composites (CNFs@BiOCl-X) were prepared by KOH activation followed by hydrothermal growth of BiOCl, aiming to develop regenerable electrodes for chloride removal in capacitive deionization systems. Alkali activation regulated the surface roughness, oxygen-containing functional groups, hydrophilicity, and BiOCl loading of CNFs, while the three-dimensional conductive network helped immobilize BiOCl nanostructures and buffer the volume variation associated with reversible Bi/BiOCl conversion. Electrochemical analyses confirmed the pseudocapacitive chloride-storage behavior of the composites, with ion removal governed by the coupled effects of BiOCl redox activity, charge transfer, and interfacial ion transport. In a fixed-electrode membrane capacitive deionization system, CNFs@BiOCl-2 exhibited the best overall performance, delivering a salt adsorption capacity of 100.44 mg g^−1^ at 1.4 V and retaining 93.17% of its desalination capacity after 35 cycles at 1.2 V. For flow-electrode capacitive deionization, the higher BiOCl-loading CNFs@BiOCl-5 showed superior utilization of active sites and achieved 94.75% NaCl removal from a 1000 mg L^−1^ solution within 3 h, with an average desalination rate of 15.48 μg cm^−2^ min^−1^ and an energy consumption of 0.88 kWh kg^−1^-NaCl. These findings demonstrate that rationally matching BiOCl loading with electrode configuration enables efficient and sustainable chloride management, offering a promising electrochemical strategy for conservation electrolytes and related desalination applications.

## 1. Introduction

Archaeological bronzes recovered from burial environments are often chemically unstable because chloride-bearing groundwater and soil electrolytes can penetrate corrosion layers over centuries [1]. Chloride ions are particularly detrimental, as they are incorporated into copper corrosion products such as cuprous chloride and subsequently trigger cyclic hydrolysis–oxidation reactions known as “bronze disease” [2]. Once exposed to air and moisture after excavation, these reactions generate acidic chloride species, sustain localized corrosion, and threaten the long-term preservation of the artifact [3]. Therefore, effective chloride removal is a central step in the stabilization of excavated bronze cultural heritage. Conventional conservation treatments mainly rely on alkaline immersion, including sodium hydroxide, sodium carbonate, or sodium sesquicarbonate solutions, to promote the diffusion of soluble chloride from corrosion layers into the surrounding electrolyte [4]. Although such methods are simple and widely used, their efficiency progressively decreases as released ions accumulate in the bath and the solution chemistry evolves during prolonged soaking. The resulting ion saturation and pH variation reduce the driving force for further chloride extraction, requiring frequent replacement of large volumes of alkaline solution. This practice increases treatment cost and generates secondary chemical waste, which conflicts with the growing demand for sustainable conservation technologies. Electrochemical dechlorination can accelerate chloride migration, but its practical application still requires careful control to avoid undesirable alteration of fragile corrosion layers or metallic substrates [5]. These limitations highlight the need for an ion-management strategy that can continuously maintain a chloride concentration gradient without repeatedly renewing the chemical bath. Capacitive deionization provides such a possibility by coupling low-voltage electrosorption with regenerable porous electrodes, enabling sustainable chloride removal from conservation electrolytes.

Capacitive deionization (CDI) has gradually become a research hotspot with its advantages of mild operating conditions, simple device structure, low energy consumption, excellent desalination performance, and no secondary pollution [6]. It is considered a new technology for desalination of seawater and wastewater that is economical, efficient, and alternative. The CDI cell system consists of a current collector and porous electrodes (fixed or flowing electrodes). An electro-adsorption process is carried out after applying voltage. Ions in the feed solution are electrostatically attracted to the opposite charged electrode and adsorbed in the double layer (EDL) formed at the electrode/water interface [7]. In ideal EDL-based systems, this adsorption is considered reversible, and ions can be released upon voltage reversal. However, in practical nanoporous electrode systems, ion adsorption behavior is considerably more complex and depends critically on the surface chemistry and the nature of the ion–surface interactions. For instance, ions interacting with functional groups at charged interfaces may exhibit either reversible electrostatic adsorption or stronger binding (e.g., chelation), which can profoundly affect the overall desalination performance and electrode regenerability [8]. Then, a desorption process is carried out after reversing the voltage, and ions are released back into the water, resulting in a saltwater flow or concentrate. Typical CDI device configurations include capacitive deionization (CDI), membrane capacitive deionization (MCDI), and flow electrode capacitive deionization (FCDI). CDI operates in batch processing mode, with water flowing between fixed electrodes for a specific time. The low desalination capacity and “co-ion discharge” effect have always constrained the practical implementation of CDI technology. Adding an ion exchange membrane (IEMs) to the cathode and anode electrode surfaces of CDI is called MCDI [9]. The addition of the membrane retains batch processing mode operation while eliminating the “co-ion discharge” effect, improving ion removal performance [10]. The emergence of FCDI introduced dynamic electrodes, where electrode materials do not need to be mixed with the binders that restrict the contact between active materials to prepare fixed electrodes. Instead, a flowing slurry or suspension is formed, and continuous desalination can be achieved by circulating the suspension through channels [11]. Different CDI devices have different requirements for materials. The development of high-performance electrode materials has become the focus of CDI technology.

Bismuth-based materials have the advantages of being green, non-toxic, low-cost, and capable of generating a large number of vacancies, and in aqueous media, bismuth can undergo specific transformation reactions with chloride ions, which gives bismuth compounds the ability to selectively trap chloride ions [12]. Nam and Choi [13] prepared nanoscale needle-like or dendritic Bi electrodes by electrodeposition, and two-dimensional (2D) BiOCl flakes could be obtained by electrochemical oxidation (1 mA cm^−2^) of Bi. Needle-like or dendritic bismuth reappeared when BiOCl was reduced to Bi, and by repeating the same oxidation/reduction process, two-dimensional (2D) BiOCl flakes and acicular/dendritic Bi could be obtained repeatedly. The reaction can be expressed by the following equation: Bi + Bi_2_O_3_ + 3Cl^−^ ↔ 3BiOCl + 3e, which means that the process is highly reversible and sustainable. This property makes bismuth compounds potentially useful in the treatment of wastewater or environments containing chloride ions. However, Bi/BiOCl electrodes produce large volume changes during charging and discharging [14], which is currently the most important difficulty to overcome for bismuth-based materials as electrode materials. In addition, bismuth-based materials as electrodes suffer from low electrical conductivity, volume expansion, and material rupture due to stress concentration [15]. To solve these problems, researchers have developed various bismuth-based composites. Currently, the development of bismuth-based composites by combining carbon isomers is the most effective and most studied direction.

Herein, we constructed a high conductivity composite (CNFs@BiOCl-X) with three-dimensional network-immobilized BiOCl nanostructures by hydrothermally loading Bi/BiOCl onto carbon nanofibers (CNFs), modified by different concentrations of alkali. The variation of BiOCl nanostructures loaded on CNFs modified by different concentrations of alkali and the effect of alkali concentration on the morphology, physical phase, and hydrophilicity of the final composites were explored through a series of characterizations. The dechlorination performance of CNFs@BiOCl-X in different CDI systems, including the fixed-electrode MCDI device and the flowing-electrode FCDI device, was also explored by comparative experiments. High-performance dechlorination of the same system materials for different concentrations of salt water in different CDI systems was realized, which effectively mitigates the volume change during the Bi/BiOCl conversion reaction and at the same time gives greater play to the advantages of the bismuth-based materials while expanding the application scenarios of the series of materials.

## 2. Experimental Section

### 2.1. Reagents and Materials

Carbon nanofibers were purchased from Jiangsu Xianfeng Nanomaterials Technology Co., Ltd. (Nangjing, China); bismuth nitrate pentahydrate (Bi (NO_3_)_3_·5H_2_O, GR, Adamas), potassium hydroxide (KOH, AR), potassium chloride (KCl, GR), ethylene glycol (EG, AR), and anhydrous ethanol (CH_3_CH_2_OH, AR) were all purchased from Greagent (Shanghai, China).

### 2.2. Materials Synthesis

Alkali modified CNFs (CNFs-X) were obtained by adding 100 mg of carbon nanofibers into 50 mL of KOH solution (concentrations of 1 M, 2 M, 3 M, 4 M, 5 M, respectively), vigorously stirring for 12 h, followed by vacuum filtration to neutrality, and freeze-drying for 24 h.

CNFs@BiOCl-X was synthesized by hydrothermal synthesis: 1.0914 g of Bi (NO_3_)_3_·5H_2_O and 0.1677 g of KCl were dissolved in 15 mL of ethylene glycol (EG) and 45 mL of ethanol, and 22.5 mg of alkali modified CNFs were added. Then, the mixed solution was thoroughly mixed by ultrasound and transferred to a 100 mL Teflon liner and autoclave, and the reaction was carried out at 160 °C for 24 h. After cooling down naturally, the reacted sample was washed with deionized water at least 5 times and freeze-dried for 24 h to obtain the modified carbon nanofiber loaded BiOCl material (CNFs@BiOCl-X). Unmodified CNFs loaded BiOCl material (CNFs@BiOCl-0) was prepared by the same method.

### 2.3. Electrochemical Test

The morphology and structure of CNFs@BiOCl-X were examined by scanning electron microscopy (SEM, Hitachi 8100, Hitachi High-Technologies Corporation, Tokyo, Japan). Powder samples were ultrasonically dispersed in ethanol, deposited onto silicon wafers, and sputter-coated with approximately 5 nm of gold after solvent evaporation. SEM imaging was performed at 10 kV with a working distance of 8–10 mm using a secondary electron detector, while EDS mapping and quantification were conducted at 15 kV, 10 mm working distance, and 60 s acquisition time. At least three regions were analyzed per sample. X-ray diffraction (XRD, Rigaku Ultimate IV, Rigaku Corporation, Akishima, Japan) patterns were recorded with Cu Kα radiation (λ = 1.5406 Å) at 40 kV and 40 mA over 10^∘^–80^∘^ at 5 min^−1^. X-ray photoelectron spectroscopy (XPS, Thermo Kalpha, Thermo Fisher Scientific, East Grinstead, United Kingdom) employed monochromatic Al Kα (1486.6 eV) under <5 × 10^−9^ mbar, with binding energies calibrated to C 1s at 284.8 eV. Contact angles (JC2000, Shanghai Zhongchen Digital Technology Apparatus Co., Ltd., Shanghai, China) were measured at room temperature using the sessile drop method on compressed pellets of each sample (prepared under 10 MPa for 2 min). A 2 μL droplet of deionized water was deposited onto the pellet surface, and the contact angle was recorded after 5 s of stabilization. The reported values are averages of at least three measurements taken at different positions on each pellet.

An electrochemical workstation (ATU85327, Wuhan LAND Electronic Co., Ltd., Wuhan, China) was used to measure and analyze the electrochemical properties of CNFs@BiOCl-X active electrodes, including cyclic voltammetry (CV) and constant-current charge/discharge tests (GCD), and information such as the charge transfer impedance of the active material was analyzed by electrochemical impedance testing (EIS, CHI660D, CH Instruments, Inc., Shanghai, China).

### 2.4. Performance Test

#### 2.4.1. MCDI Desalination Tests

The active material (CNFs@BiOCl-X), conductive additive (acetylene black, CB), and binder (polyvinylidene difluoride, PVDF) were thoroughly mixed in the mass ratio of 8:1:1, and an appropriate amount of solvent (N-methylpyrrolidone (NMP)) was added to the mixed powder. An electrode slurry was obtained after stirring for 12 h, the slurry was coated on the carbon paper, and dried to obtain a 2 cm × 2 cm fixed electrode.

The fixed electrode (MCDI) mode device was symmetrically assembled from two acrylic base plates, two silicone spacers, two silver sheet electrodes and a CNFs@BiOCl-X electrode, and a sheet of anion-exchange membrane (AEM, membrane surface resistance (MSR) ≤ 2.7 Ω cm^2^ (0.5 M NaCl, 25 °C) on one side with an activated carbon electrode and a sheet of cation-exchange membrane (CEM, MSR ≤ 5.5 Ω cm^2^ (0.5 M NaCl, 25 °C)), Hangzhou Huafilm Technology Co., Hangzhou, China) on the other side, as well as a chamber in the middle. The CDI system consisted of a power supply, feed liquid container, CDI unit, conductivity meter, and peristaltic pump. The NaCl removal test was performed in constant voltage mode with cut-off voltages of 1 V, 1.2 V, and 1.4 V. A constant voltage was applied to the cathode and anode by a blue power test system, and the voltage/current data were recorded in real time. The simulated brine for the test was 500 mg L^−1^ NaCl solution with a volume of 40 mL and a flow rate of 20 mL min^−1^. The conductivity of NaCl was linearly related to its concentration, and the conductivity change of the simulated brine was read in real time by a conductivity meter (LeiCi DDSJ-319L, INESA Scientific Instrument Co., Ltd., Shanghai, China), with a 10 s interval between the data recordings.

The desalination performances of the MCDI device were evaluated and estimated by NaCl adsorption capacity (SAC, mg g^−1^), average salt adsorption rate (ASAR, mg g^−1^ min^−1^), energy consumption (EC, kWh kg^−1^-NaCl) equation according to Equations (1)–(3):

(1) $$\mathrm{SAC}=\frac{(C_{0,\mathrm{NaCl}}-C_{t,\mathrm{NaCl}})\times V}{m_{\mathrm{total}}}$$

(2) $${\mathrm{ASAR}}_{\mathrm{MCDI}}=\frac{{\mathrm{SAC}}_{t}}{t_{c}}$$

(3) $$\mathrm{EC}=\frac{{u\times \int i\;\mathrm{dt}}_{t}}{3.6\times (C_{0,\mathrm{NaCl}}-C_{t,\mathrm{NaCl}})\times V}$$

where $C_{0,\mathrm{NaCl}}$ (mg L^−1^) and $C_{t,\mathrm{NaCl}}$ (mg L^−1^) are the initial and final NaCl concentrations, respectively, V (L) the volume of the NaCl feed solution, $m_{\mathrm{total}}\;$(g) is the total mass of the two electrodes, $t_{c}$ (min) and t (s) are the charge time, u (V) is a constant applied voltage during the desalination process, and i (A) is the instantaneous charging current.

#### 2.4.2. FCDI Desalination Tests

The active material (CNFs@BiOCl-X) was mixed with carbon black (CB) at a mass ratio of 4:1, and then 50 mL of NaCl solution with the same concentration as that of the solution to be treated was added, and a flowing electrode slurry was formed by stirring for 12 h. During this period, in order to promote complete dissolution of the hydrophobic CB, the surfactant sodium dodecyl sulfate (SDS) was added in appropriate amounts.

The flow capacitor deionization device was symmetrically assembled from a base plate, silica gel pad, collector, anion exchange membrane, chamber, and cation exchange membrane, with the cation membrane in the middle of the two chambers separating the two chambers into a fresh water chamber and a concentrated water chamber. The collector was a graphite plate on which 13 flow channels of 50 mm length, 3 mm width, 2 mm depth, and 2 mm spacing were carved. The flowing electrode slurry was pumped in via the bottom plate and flowed well in the flow channels. The experiment adopted the single cycle (SC) mode with an additional brine separation channel [16] in FCDI, in which the flowing electrode slurry (CNFs@BiOCl-X) was pumped into the collector flow channel, and the electrode slurry flowed through the cathode and anode chambers and finally flowed into the same electrode tank, where it was regenerated spontaneously, and then pumped into the cathode chamber again to recycle. This facilitated the elimination of back-voltage due to changes in the H^+^ or OH^−^ content on both sides of the membrane [17,18].

The desalination performances of the FCDI device were evaluated by average desalination rate (ASAR, mg cm^2^ min^−1^), removal efficiency (RE, %), and the charge efficiency (Λ, %) equation according to Equations (4)–(6). The energy consumption (EC, kWh kg^−1^-NaCl) equation is consistent with Equation (3):

(4) $${\mathrm{ASAR}}_{\mathrm{FCDI}}=\frac{{(C_{0,\mathrm{NaCl}}-C_{t,\mathrm{NaCl}})\times V}_{t}}{A\times t_{c}}$$

(5) $$\mathrm{RE}=\frac{C_{0,\mathrm{NaCl}}-C_{t,\mathrm{NaCl}}}{C_{0}}$$

(6) $$\Lambda =\frac{{\mathrm{SAC}\times F\times m}_{t}}{58.5\times 1000\times \int i\;\mathrm{dt}}$$

where A (cm^2^) is the contact area between the flow electrode and the ion exchange membrane, F is the Faraday charge constant (96,485 C mol^−1^), and *m_t_* (g) is the total mass of the active material in the flow electrode. The other symbols have the same meaning as the symbols in the equation in Section 2.4.1.

All MCDI desalination experiments were performed with four cycles at each cutoff voltage (1.0, 1.2, and 1.4 V), corresponding to a total of 12 cycles per material. The reported SAC, ASAR, and EC values represent the mean ± standard deviation (SD) calculated from these four replicate cycles.

## 3. Results and Discussion

### 3.1. Morphology and Structure of CNFs@BiOCl-X

**Figure 1:**
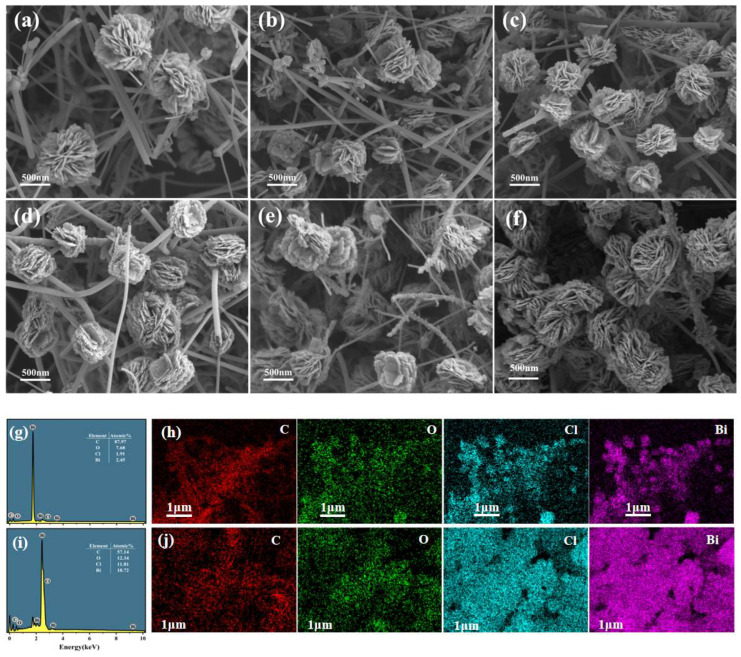
SEM patterns of (**a**) CNFs@BiOCl-0, (**b**) CNFs@BiOCl-1, (**c**) CNFs@BiOCl-2, (**d**) CNFs@BiOCl-3 (**e**) CNFs@BiOCl-4 (**f**) CNFs@BiOCl-5; EDS energy spectrum of (**g**) CNFs@BiOCl-2 and (**i**) CNFs@BiOCl-5; EDS mapping of (**h**) CNFs@BiOCl-2 and (**j**) CNFs@BiOCl-5. EDS spectra were normalized to the carbon Kα peak for comparative purposes.

The samples are denoted as CNFs@BiOCl-X, where X (=0, 1, 2, 3, 4, or 5) represents the molar concentration of KOH (0, 1, 2, 3, 4, or 5 M) used for alkali activation of the carbon nanofibers prior to BiOCl loading; X = 0 corresponds to unactivated CNFs. Scanning electron microscopy (SEM) was used to characterize the morphology and structure of non-alkali-activated and different concentrations of alkali-activated CNFs@BiOCl, as shown in Figure 1a–f. CNFs@BiOCl-X presents a three-dimensional network structure consisting of numerous nanofibers with diameters of about 50–150 nm, and the BiOCl sheets stacked into spherical flowers with diameters of about 500–600 nm are uniformly loaded in this three-dimensional network and are tandemly connected by carbon nanofibers. With the enhancement of the KOH concentration used for activation, the surface of the carbon nanofibers becomes rougher with more pronounced concave undulations and pore structures. It indicates that alkali activation has successfully changed the surface morphology of the CNFs to have more active sites for the loading and growth of Bi^3+^ [19]. Therefore, it can be seen that the modified carbon nanofibers with higher alkali concentration are loaded with more BiOCl nanostructures of greater number and density. Among them, the BiOCl nanoflowers of CNFs@BiOCl-2 have become significantly denser and more numerous compared to the original material. The three-dimensional network structure can not only immobilize BiOCl to avoid agglomeration and alleviate the volumetric phase transition problem during Bi/BiOCl conversion but also form a conductive highway to enable rapid electron transfer between active materials, so that more active materials can participate in the reaction and enhance the dechlorination performance and stability of the materials.

Figure 1g–j demonstrate the EDS energy spectra and EDS mapping of CNFs@BiOCl-2 and CNFs@BiOCl-5, which shows that the four elements, C, O, Cl, and Bi, are uniformly distributed in the materials with elemental ratios that are basically the same as those with their structures. This further indicates that the BiOCl structure is successfully loaded on the alkali-modified CNFs. With increasing alkali concentration, the relative content ratio of O, Cl, and Bi all increased significantly in the EDS spectra, suggesting that higher KOH activation concentrations led to greater BiOCl loading on the CNFs. However, it should be noted that EDS analysis provides only semi-quantitative information on elemental composition. While the observed increase in BiOCl-related signals correlates with enhanced electrochemical performance up to an optimal loading (CNFs@BiOCl-2), the subsequent decline in performance at higher loadings (CNFs@BiOCl-3, -4, and -5) cannot be attributed solely to the increased BiOCl content. As discussed below, other interdependent factors—including the degree of BiOCl nanoflower densification, the resulting restriction of ion accessibility, the surface hydrophilicity, and the structural stability against volume expansion during the Bi/BiOCl conversion—also play critical roles in determining the overall electrochemical and desalination performance.

**Figure 2:**
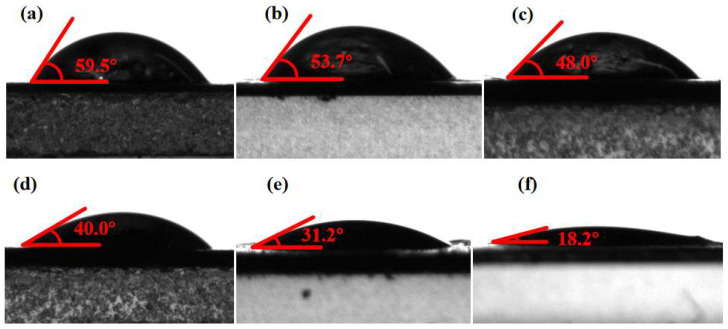
Water contact angle of (**a**) CNFs@BiOCl-0, (**b**) CNFs@BiOCl-1, (**c**) CNFs@BiOCl-2, (**d**) CNFs@BiOCl-3, (**e**) CNFs@BiOCl-4 and (**f**) CNFs@BiOCl-5.

Figure 2 demonstrates the water contact angle of CNFs@BiOCl-X materials, and the synthesized materials obviously possessed a smaller water contact angle with the enhancement of the pretreatment alkali concentration. This indicates that the alkali modification makes the CNFs surface possess more oxygen-containing functional groups and improves the hydrophilicity of the material, which is conducive to lowering the ionic mass transfer resistance at the solid–liquid interface and effectively improving the ionic transport efficiency; this contributes to the enhancement of the adsorption rate the and dechlorination performance of the composites. Although the higher concentration of alkali activation resulted in higher hydrophilicity, the BiOCl nanostructures, which gradually became thick and bulky, were not conducive to the stabilization of the stationary electrodes. Therefore, lower concentrations of alkali activated CNFs loaded with BiOCl are more favorable for the use of fixed electrodes for desalination.

**Figure 3:**
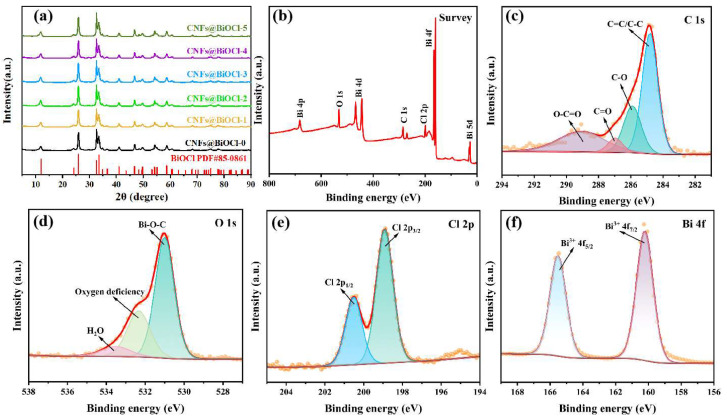
(**a**) XRD spectra of CNFs@BiOCl-X; XPS spectra of CNFs@BiOCl-5. (**b**) Survey spectrum, and (**c**) C 1s, (**d**) O 1s, (**e**) Cl 2p, (**f**) Bi 4f.

The crystal structure of CNFs@BiOCl-X materials was verified by X-ray diffraction (XRD) analysis, as shown in Figure 3a; the composite material showed more obvious diffraction peaks at 2θ = 11.999°, 25.877°, 32.525°, 33.468°, 40.907°, 46.661°, and 58.634°, and though the comparison and the peak positions of the BiOCl standard card (PDF#85-0861) are basically the same, it indicates the successful loading of the BiOCl structure on the CNFs. Moreover, all the samples had similar XRD patterns, which indicated that BiOCl was successfully loaded on the surface of CNFs in all the samples, and only the compositional nanostructures were different.

The surface composition and chemical state of the materials were studied using XPS. The full spectrum, as shown in Figure 3b, shows the presence of Bi, Cl, O, and C. The peaks located at 284.8, 286.0, 287.0, and 289.1 eV in the C 1s spectrum as shown in Figure 3c are associated with C=C, C-O, C=O, and O-C=O [20,21]. In the O 1s spectrum shown in Figure 3d, the peaks at 531.0 and 532.3 eV correspond to Bi-O-C bonds and imperfect lattice defect oxygen (OM), respectively, and the other peak is located at 533.5 eV, which belongs to water adsorbed by the material [22]. As shown in the Cl 2p spectrum in Figure 3e, two peaks located at 198.9 and 200.5 eV can be assigned to Cl 2p_3/2_ and Cl 2p_1/2_, proving the presence of Cl^−^ [22]. The peaks in the Bi 4f spectrum, shown in Figure 3f, consist of Bi^3+^ 4f_7/2_ and Bi^3+^ 4f_5/2_ located at 160.2 and 165.5 eV [23].The presence of the characteristic peaks of Bi^3+^, Cl^−^, and Bi-C-O indicates the successful loading of BiOCl on the carbon nanofibers.

### 3.2. Electrochemical Performance of CNFs@BiOCl-X

**Figure 4:**
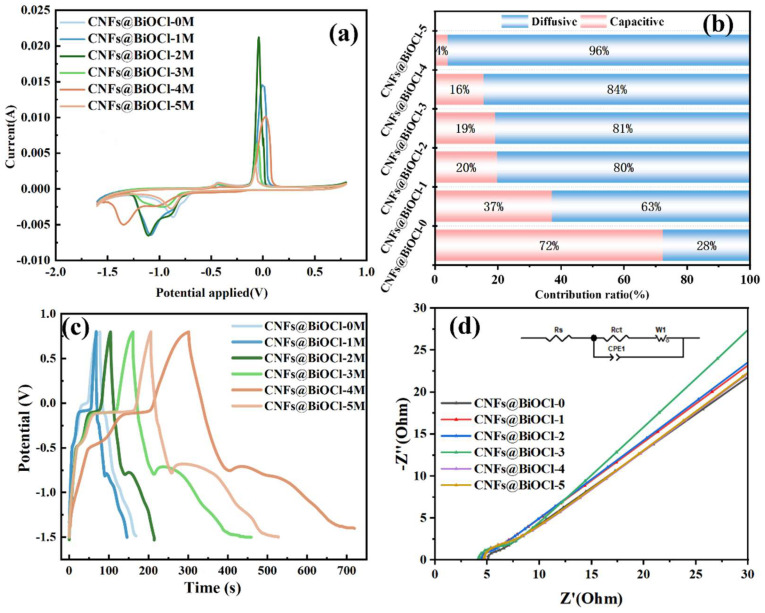
(**a**) Cyclic voltammetry curve of CNFs@BiOCl-X at 5 mV s^−1^ scan rate; (**b**) comparison of diffusion/capacitance contribution of CNFs@BiOCl-X at 10 mV s^−1^ scan rates; (**c**) constant current charge–discharge curve of CNFs@BiOCl-X at 1 A g^−1^ current density; (**d**) based on electrochemical impedance spectroscopy (EIS), measurements with the frequency range from 100 kHz to 0.01 Hz of CNFs@BiOCl-X.

The electrochemical properties of CNFs@BiOCl-X were determined using a three-electrode system in 1.0 mol L^−1^ NaCl solution. Figure 4a demonstrates the CV curves of CNFs@BiOCl-X at a scan rate of 5 mV s^−1^, with a potential window of −1.6–0.8 V (with respect to the Ag/AgCl electrodes). The presence of oxidizing peaks around 0 V and reducing peaks at −1 V for all the materials within the test voltage window corresponds to the reversible reaction of Bi^3+^↔Bi^0^, suggesting that electrochemical processes induced by the Bi/BiOCl conversion induced pseudocapacitive behavior. Among them, CNFs@BiOCl-2 showed the largest peak current response, indicating a better charge transfer capability and higher electrochemical reaction kinetics—which is favorable for improving the desalination performance—followed by CNFs@BiOCl-1 and CNFs@BiOCl-4. Figure 4c demonstrates the constant-current charge–discharge (GCD) curves of CNFs@BiOCl-X at a current density of 1 A g^−1^. The GCD curves of all materials show a clear plateau in the charging and discharging stages, which basically corresponds to the redox peaks appearing in the CV curves above, indicating that the Faraday reaction occurs in the materials, which corresponds to the reversible redox reaction of Bi^3+^↔Bi^0^. The voltage plateau can be seen to become longer with the increase in the alkali concentration of the modified carbon nanofibers and the increase in the amount of the loaded BiOCl, indicating that the voltage plateau becomes longer at a lower current density—the more active substances involved in the redox reaction, the longer the time required for the completion of the oxidation/reduction reaction. Alkali concentration increased from 1 M to 4 M corresponding to the electrode material. The charge–discharge time in the GCD curve gradually increased, indicating that its specific capacity in the ensuing increase was more conducive to the storage of ions. The alkali concentration is further increased to 5 M and the charge–discharge time in the GCD curve compared to 4 M becomes shorter, because at this time the BiOCl nanosheets structure is gradually changed to a block and the volumetric phase transition that occurs in the testing process of Bi/BiOCl seriously affects the electrode stability, resulting in a decrease in electrochemical performance. This is consistent with the fact that the percentage of diffusion control of CNFs@BiOCl-X material shown in Figure 4b increases with the elevation of the alkali concentration of the treated CNFs and the contribution of the diffusion-controlled process of CNFs@BiOCl-5 dominates the overall electrochemical behaviors when the alkali concentration is increased to 5 M. The diffusion-controlled process of CNFs@BiOCl-5 is also shown in Figure 4c.

To further understand the internal resistance and ion transport behavior of each material, electrochemical impedance experiments were performed on the materials, as shown in Figure 4d. The EIS spectra are mainly composed of quasi semicircles in the high frequency region and quasilinear lines in the low frequency region. The size of the diameter of the quasi semicircle in the high frequency region can be used to determine the material’s charge transfer resistance Rct—the larger the diameter, the larger the Rct. In addition, the slope of the quasilinear line region can be used to determine the magnitude of the material’s Warburg impedance—the larger the slope, the smaller the Rw [24]. With the rise of alkali concentration, the diameter of the quasi semicircle in the high-frequency region increases and then decreases with the increase of alkali concentration, and the diameter is the largest and Rct is the largest at CNFs@BiOCl-3; the linear curvature in the low-frequency region increases and then decreases, and the slope is the largest and Rw is the smallest at CNFs@BiOCl-3. The modification of alkali increases the pore structure of CNFs, so that the composites possess a faster redox reaction rate at the electrode–electrolyte interface, which is conducive to the improvement of the desalination ability of the materials. The charge transfer resistances (Rct) of all materials are comparable, with CNFs@BiOCl-3 slightly higher than CNFs@BiOCl-2, yet the latter shows superior CV/GCD performance due to an optimal balance of BiOCl loading and nanostructural openness. Excessive loading (≥3 M) causes nanoflower densification, restricting ion access and exacerbating volume expansion during Bi/BiOCl conversion, which undermines structural integrity and active-material utilization more than the modest Rct increase. Thus, CNFs@BiOCl-2 achieves the best overall performance by maximizing pseudocapacitive activity while maintaining efficient ion transport and structural stability.

### 3.3. Desalination Performance in MCDI

**Figure 5:**
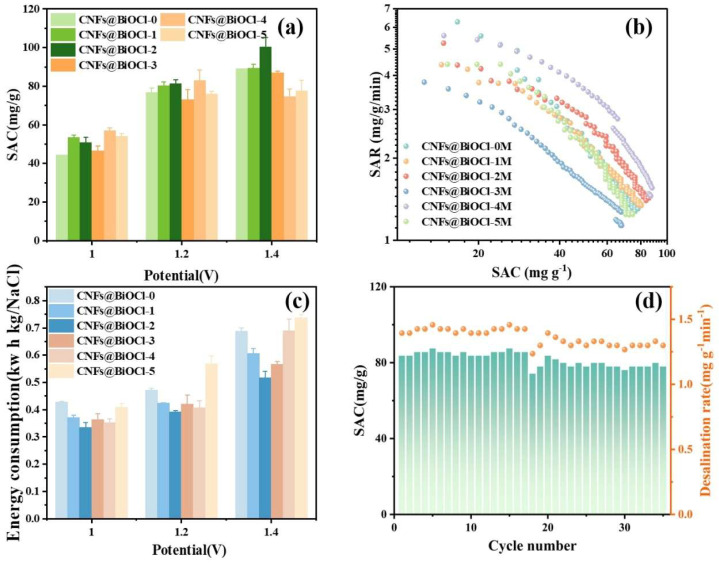
(**a**) Desalting capacity comparison, (**b**) Ragone diagram, (**c**) energy consumption comparison plot at different cutoff voltages of CNFs@BiOCl-X, (**d**) desalting long cycle test.

The operating voltage is crucial to the performance of CDI, and in this experiment, the desalination performance of CNFs@BiOCl-X was tested by adopting the constant voltage mode for a total of 12 cycles at three cutoff voltages, namely, 1 V, 1.2 V, and 1.4 V. As shown in Figure 5a, at lower voltages (1.0–1.2 V), the desalination capacities of CNFs@BiOCl-X all increased with increasing voltage and were generally superior to that of CNFs@BiOCl-0. CNFs@BiOCl-4 exhibited the maximum desalting capacity (83.12 mg g^−1^) at 1.2 V, slightly higher than that of CNFs@BiOCl-2. However, given the overlap of their error bars shown in Figure 5a, this difference should be considered marginal rather than definitive. The Ragone diagram of CNFs@BiOCl-X at 1.2 V is shown in Figure 5b. From the energy consumption plots at different operating voltages shown in Figure 5c, CNFs@BiOCl-4 also exhibited low energy consumption at low voltages, only slightly higher than that of CNFs@BiOCl-2. This corresponds to the electrochemical performance described above. However, with a further increase in voltage to 1.4 V, the desalination performance of CNFs@BiOCl-4 showed substantial degradation accompanied by a sharp rise in energy consumption, suggesting that the reaction became more rapid with an increase in the applied voltage; the denser BiOCl nanostructures underwent more pronounced volume changes, disrupting the composite structure and leading to decreased electrode performance. CNFs@BiOCl-5, due to the denser and thicker loaded BiOCl nanostructures and the larger volume change in the reaction, not only showed a similar performance decay as that of CNFs@BiOCl-4 but also the performance did not continue to increase due to the increase in the BiOCl loading at low voltages (1.0–1.2 V). At the same time, the detachment of the active material due to its poor stability also increased the electron mass transfer resistance, and the energy consumption also showed a significant increase. It indicates that the CNFs@BiOCl-4 and CNFs@BiOCl-5 materials with higher BiOCl active substance loadings are not suitable for desalination as fixed electrodes for fixed capacitive deionization.

On the contrary, the desalting capacity of CNFs@BiOCl-2 can increase with voltage, and the desalting performance at low voltage (1.0–1.2 V) is only slightly lower than that of CNFs@BiOCl-4. It shows the highest performance at higher voltage (1.4 V), up to 100.44 mg g^−1^, and the lowest energy consumption at different voltages, which suggests that the structure of the CNFs@BiOCl-2 is the most favorable for charge transfer and that ion transport is most favorable. As shown in Figure 5d, the desalination performance of CNFs@BiOCl-2 can still be maintained at 93.17% after 35 charge/discharge cycles at 1.2 V. The average desalting capacity was 81.8 mg g^−1^, and the average desalting rate was 1.36 mg g^−1^ min^−1^ of 35 cycles, indicating its good stability as a fixed electrode. The retention of desalination performance suggests that the three-dimensional CNF network effectively mitigates the structural stress associated with the volume changes during reversible Bi/BiOCl conversion. However, we acknowledge that this proposed buffering mechanism is inferred indirectly from the electrochemical stability data rather than directly demonstrated by post-cycling characterization. Future work will include systematical post-cycling SEM, XRD, and XPS analyses to provide direct evidence of the structural and chemical stability of the CNF-immobilized BiOCl nanostructures after prolonged cycling. Collectively, the MCDI results demonstrate that the CNF@BiOCl-2 electrode offers a competitive salt adsorption capacity and outstanding cycling stability among BiOCl-based fixed-electrode systems, effectively addressing the trade-off between active-site utilization and structural integrity that has limited prior bismuth-based CDI electrodes. Nevertheless, compared with other materials, CNFs@BiOCl-2, with appropriate BiOCl nanosheet loading, has a structure that exhibits a looser nanoflower, which better balances the relationship between BiOCl loading and nanostructure and can make full use of the conductive network composed of CNFs. It also has excellent desalination performance and cycling stability as a stationary electrode, which gives it potential for long-term use in practical applications.

### 3.4. Desalination Performance in FCDI

Although CNFs@BiOCl-4 and CNFs@BiOCl-5 obtained superior electrochemical performance and better hydrophilicity due to the loading of more BiOCl, the denser structure produced more obvious deformation during the conversion reaction, which greatly affected the stability of the stationary electrode and was not as suitable as that of CNFs@BiOCl-2 as a stationary electrode for CDI desalination. However, the three-dimensional network composed of carbon nanofibers has good electrical conductivity, certain mechanical properties, and good hydrophilicity, which meets the requirements of active materials for flow electrodes. Therefore, we believe that if it is used as a flow electrode, it can avoid the problem of volume change in the reaction and can more fully utilize the hydrophilicity of the material and the active sites of the loaded BiOCl; this can bring out the advantages of the material of this system to a greater extent and expand the application scenarios. For more obvious comparison and validation of the above points, we prepared CNFs@BiOCl-2—which has a better overall performance in fixed electrodes—and CNFs@BiOCl-5—which has the highest BiOCl loading and the best hydrophilicity—as a flow electrode slurry in FCDI for the test and comparison of the desalination performance.

**Figure 6:**
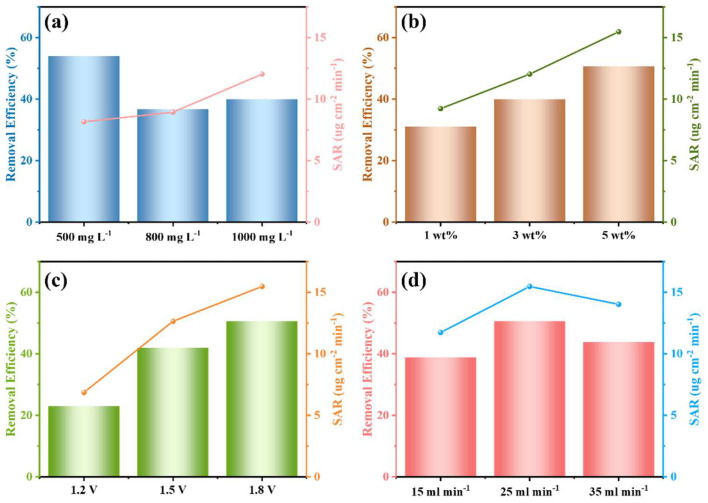
Desalting removal rate and desalination removal efficiency of CNFs@BiOCl-5 (**a**) at different influent concentrations; (**b**) under different electrode loadings; (**c**) at different operating voltages; (**d**) at different electrode flow rates.

First, in order to obtain the best performance of the materials in the FCDI system, we conducted experiments on the effects of four parameters, namely, influent water concentration, electrode loading, operating voltage, and slurry flow rate, on the desalination effect of the FCDI system during one hour of operation time. When the influent concentration was increased from 500 mg L^−1^ to 1000 mg L^−1^, the salt removal efficiency (RE) and desalination rate (ASAR) in the FCDI system are shown in Figure 6a, and the desalination rate increased significantly from 8.16 to 12.03 μg cm^−2^ min^−1^. Meanwhile, the system’s current response was significantly strengthened with the increase of influent water concentration as shown in Figure S1a,b, and the salt solution concentration decreased more at a fixed time. As shown in Figure S1c, the change in energy consumption with the increase in feed water concentration was small—0.77, 0.83, and 0.86 kWh kg^−1^-NaCl, respectively—and the change in the concentration of treatment solution did not have a significant effect on the energy consumption of the system. Combining the desalination capacity and energy consumption changes, the higher concentration of solution can effectively reduce the ion transmembrane migration resistance, while the FCDI system also shows excellent treatment capacity for higher concentration of salt solution.

When the electrode loading was increased from 1 wt% to 5 wt%, the salt removal efficiency (RE) and the desalination rate (ASAR) in the FCDI system are shown in Figure 6b, where the rate of desalination was increased from 9.22 to 15.48 μg cm^−2^ min^−1^. At the same time, the rate of desalination increased from 31.06% to 50.60%. As demonstrated in Figure S2a,b in the Supporting Information, both the system current and concentration reduction rate showed positive correlations with electrode loading, with higher loadings resulting in greater current generation and faster salt solution concentration decrease at fixed time intervals. Notably, the energy consumption remained relatively stable despite the increasing electrode loading (Figure S2c) rising modestly from 0.69 kWh kg^−1^-NaCl at 1 wt% to 0.86 kWh kg^−1^-NaCl at 3 wt% and maintaining comparable levels (0.88 kWh kg^−1^-NaCl) even when the loading was further increased to 5 wt%. This suggests that higher flow electrode loading not only provides more ion adsorption sites but also makes the contact between active materials closer, accelerating the charge transfer rate and improving the conductivity of the solution [25,26]. However, the flowing electrode slurry is heterogeneous and rheological, and the material will inevitably deform in the process of continuous cyclic flow, which leads to a reduction of the charge connection between the active materials within the material [27]. The conversion reaction of Bi/BiOCl inherently produces a large volume change, and the part that is not tightly connected to the carbon nanofibers is easy to detach from the structure during the reaction process, while a higher electrode loading helps to enable more active material to participate in the reaction, counteracting the effect of some of the material detachment. The highest ASAR is 15.48 μg cm^−2^ min^−1^ at 5 wt% loading concentration, and the energy consumption is relatively low, so that 5 wt% CNFs@BiOCl-5 electrode loading may be a more suitable electrode loading for the FCDI system.

The salt removal efficiency (RE) and desalination rate (ASAR) in the FCDI system were 22.98, 41.93, and 50.60% and 6.86, 12.65, and 15.48 μg cm^−2^ min^−1^ at 1.2, 1.5, and 1.8 V, respectively, and the desalination performance exhibited a substantial increase with increasing voltage (Figure 6c). When the operating voltage is increased from 1.2 V to 1.8 V, the higher voltage possesses a larger system response current (Figure S3a) and the decrease in salt solution concentration is significantly larger (Figure S3b). This is because a higher voltage can bring a larger driving force in the desalination system. At the same time, the increase in voltage inevitably brings about an increase in energy consumption; as shown in Figure S3c, the energy consumption increases when the voltage is increased from 1.2 V (0.67 kWh kg^−1^-NaCl) to 1.5 V (0.85 kWh kg^−1^-NaCl) and then slows down significantly when the voltage is continued to 1.8 V (0.88 kWh kg^−1^-NaCl).

However, the higher desalination performance of the FCDI system was demonstrated at a slurry flow rate of 25 mL min^−1^, which is due to the fact that too fast a slurry flow rate makes the reaction incomplete, resulting in a decrease in the removal rate at a flow rate of 35 mL min^−1^ (Figure 6d). Figure S4a (Supporting Information) shows that the faster flow rate possesses a greater current response, which is because the faster slurry flow rate exacerbates the collisions between the electrode particles and results in a faster electron transport and an increased charge transfer rate.

**Figure 7:**
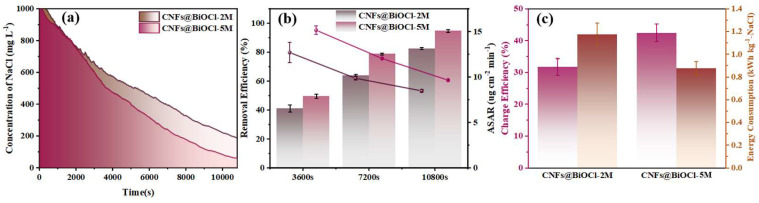
(**a**) Variation of salt solution concentration in fresh water chamber, (**b**) desalination removal efficiency and desalination removal rate, (**c**) charging efficiency and energy consumption of CNFs@BiOCl-2 and CNFs@BiOCl-5.

The FCDI performance of CNFs@BiOCl-2 and CNFs@BiOCl-5 was systematically compared under optimized operational parameters (feed concentration: 1000 mg L^−1^, electrode loading: 5 wt%, applied voltage: 1.8 V, slurry flow rate: 25 mL min^−1^). As shown in Figure 7a, CNFs@BiOCl-2 initially demonstrated comparable desalination rates to CNFs@BiOCl-5 due to its balanced composite structure, despite the latter’s superior hydrophilicity and higher BiOCl loading. However, as the reaction progressed, the conductive carbon black facilitated continuous activation of the BiOCl structures in the electrode slurry, enabling greater participation of BiOCl in the desalination process. This activation mechanism resulted in significantly enhanced performance for CNFs@BiOCl-5, achieving a 94.75% salt removal rate for 1000 mg L^−1^ NaCl solution after 3 h—a marked improvement over CNFs@BiOCl-2’s 82.67% removal rate (Figure 7b). The performance superiority of CNFs@BiOCl-5 was further corroborated by the energy consumption analysis in Figure 7c, demonstrating lower specific energy consumption (0.88 kWh kg^−1^-NaCl) compared to CNFs@BiOCl-2 (1.18 kWh kg^−1^-NaCl). CNFs@BiOCl-5 obtained superior electrochemical properties due to more BiOCl loading and better hydrophilicity due to higher concentration of alkali activation. In the flow electrode system, it remains unaffected by electrode deformation caused by the volumetric changes during reactions, enabling more efficient utilization of the material’s hydrophilic properties and the active sites of the loaded BiOCl. These FCDI results confirm that the same material platform, when deployed as a flow electrode, can overcome the volumetric-stability constraints of fixed-electrode configurations and achieve efficient, sustained desalination of higher-salinity feedwaters, a capability that conventional alkaline desalination methods cannot provide without frequent solution replacement. Consequently, the advantages of this material system are maximized.

It should be noted, however, that the FCDI experiments were conducted over a relatively short duration (3 h), and long-term cycling data for the CNFs@BiOCl-5 flow electrode are not yet available. Given the concerns about material detachment and volume expansion during continuous flow, the long-term structural integrity and reusability of CNFs@BiOCl-5 in flowing slurries require systematic evaluation. Future work will focus on extended cycling tests to validate the sustainable and regenerable nature of the CNFs@BiOCl flow-electrode system.

## 4. Conclusions

In this work, alkali-modified carbon nanofibers were used to construct CNFs@BiOCl-X composites with three-dimensional conductive networks for chloride removal in capacitive deionization systems. KOH activation regulated the surface roughness, hydrophilicity, and BiOCl loading on CNFs, thereby tailoring the balance between ion transport, charge transfer, and structural stability. The CNF network effectively immobilized BiOCl nanostructures, improved electrical conductivity, and mitigated the structural stress associated with the reversible Bi/BiOCl conversion reaction. In the fixed-electrode MCDI system, CNFs@BiOCl-2 delivered the best overall performance, achieving a salt adsorption capacity of 100.44 mg g^−1^ at 1.4 V and retaining 93.17% of its desalination capacity after 35 cycles. Higher BiOCl loadings enhanced active-site density and hydrophilicity, but excessive growth of dense BiOCl structures compromised the fixed-electrode stability because of volume-change-induced deformation. In contrast, the FCDI configuration alleviated this limitation by allowing the active material to operate as a flowing electrode, enabling more efficient utilization of highly loaded BiOCl. Under optimized conditions, CNFs@BiOCl-5 achieved 94.75% removal of 1000 mg L^−1^ NaCl within 3 h, with a desalination rate of 15.48 μg cm^−2^ min^−1^ and a low energy consumption of 0.88 kWh kg^−1^-NaCl. These results demonstrate that matching the BiOCl loading and electrode configuration is critical for maximizing chloride-removal performance. Overall, CNFs@BiOCl composites provide a promising and sustainable electrode platform for continuous chloride management in conservation electrolytes and related desalination applications. Future work will incorporate quantitative determination of the BiOCl loading using techniques such as inductively coupled plasma optical emission spectroscopy (ICP-OES) or thermogravimetric analysis (TGA), combined with systematic variation of the synthesis parameters to decouple the effects of loading, morphology, and site accessibility on the desalination performance.

## Data Availability

The original contributions presented in this study are included in the article/Supplementary Materials. Further inquiries can be directed to the corresponding authors.

## Supplementary Materials

The following supporting information can be downloaded at: https://www.mdpi.com/article/10.3390/nano16150907/s1, Figure S1: Effect of influent NaCl concentration on the FCDI desalination performance of CNFs@BiOCl-5. (a) Current response profiles of the FCDI system at different influent NaCl concentrations. (b) Time-dependent variation of NaCl concentration in the desalination chamber. (c) Specific energy consumption at different influent NaCl concentrations; Figure S2: Effect of electrode loading on the FCDI desalination performance of CNFs@BiOCl-5. (a) Current response profiles of the FCDI system at different electrode loading. (b) Time-dependent variation of electrode loading in the desalination chamber. (c) Specific energy consumption at different electrode loading; Figure S3: Effect of voltage on the FCDI desalination performance of CNFs@BiOCl-5. (a) Current response profiles of the FCDI system at different voltages. (b) Time-dependent variation of voltage in the desalination chamber. (c) Specific energy consumption at different voltage; Figure S4: Effect of slurry flow rate on the FCDI desalination performance of CNFs@BiOCl-5. (a) Current response profiles of the FCDI system at different slurry flow rate. (b) Time-dependent variation of slurry flow rate in the desalination chamber. (c) Specific energy consumption at different slurry flow rates.
