# Supplementary material for: Sustainable Chloride Removal from Conservation Electrolytes Using Alkali-Activated Carbon Nanofiber-Supported BiOCl in Capacitive Deionization
Source: Nanomaterials (Basel). 2026 Jul 24;16(15):907. doi: 10.3390/nano16150907 (PMC13468657; doi:10.3390/nano16150907)
Supplement: Supplementary file 1 [file nanomaterials-16-00907-s001.zip › nanomaterials-4419925-supplementary.pdf]

Sustainable Chloride Removal from Conservation Electrolytes Using Alkali-Activated Carbon Nanofiber-Supported BiOCl in Capacitive Deionization

Aoze Li [1,2], Fanghui Pan [1,*], Liping Sun [1,3], Mengying Xu [4,5], Ran Zhang [4,5], Fei Yu [1,6,*] and Jie Ma [1,3,7]

[1] Water Resources and Water Environment Engineering Technology Center, Xinjiang Key Laboratory of Synthesis and Application of Carbon Nanomaterials, School of Civil Engineering, Kashi University, Kashi 844000, China; aozeli@umass.edu (A.L.); m230501321@st.shou.edu.cn (L.S.); jma@tongji.edu.cn (J.M.)
[2] Stockbridge School of Agriculture, University of Massachusetts, Amherst, MA 01003, USA
[3] Research Center for Environmental Functional Materials, State Key Laboratory of Water Pollution Control and Green Resource Recycling, College of Environmental Science and Engineering, Tongji University, Shanghai 200092, China
[4] National Museum of China, Beijing 100006, China; xumengying@chnmuseum.cn (M.X.); zhangran@chnmuseum.cn (R.Z.)
[5] Key Scientific Research Base of Metal Conservation (National Museum of China), National Cultural Heritage Administration, Beijing 100006, China
[6] College of Oceanography and Ecological Science, Shanghai Ocean University, No 999, Huchenghuan Road, Shanghai 201306, China
[7] Shanghai Institute of Pollution Control and Ecological Security, Shanghai 200092, China
* Correspondence: pfh2024@ksu.edu.cn (F.P.); f-yu@shou.edu.cn (F.Y.)

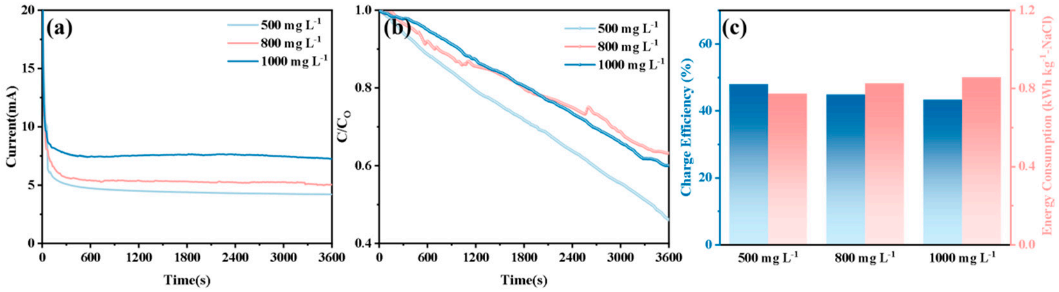

Figure S1 Effect of influent NaCl concentration on the FCDI desalination performance of CNFs@BiOCl-5. (a) Current response profiles of the FCDI system at different influent NaCl concentrations. (b) Time-dependent variation of NaCl concentration in the desalination chamber. (c) Specific energy consumption at different influent NaCl concentrations.

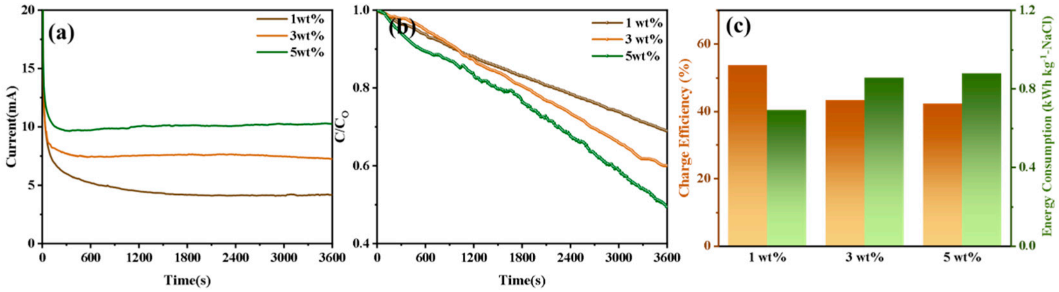

Figure S2 Effect of electrode loading on the FCDI desalination performance of CNFs@BiOCl-5. (a) Current response profiles of the FCDI system at different electrode loading. (b) Time-dependent variation of electrode loading in the desalination chamber. (c) Specific energy consumption at different electrode loading.

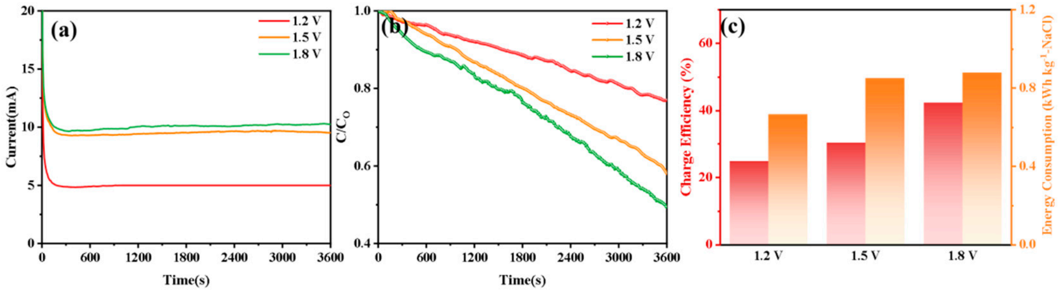

Figure S3 Effect of voltage on the FCDI desalination performance of CNFs@BiOCl-5. (a) Current response profiles of the FCDI system at different voltage. (b) Time-dependent variation of voltage in the desalination chamber. (c) Specific energy consumption at different voltage.

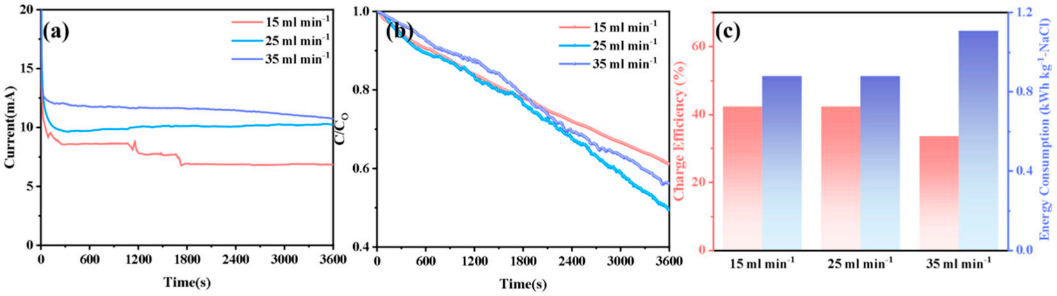

Figure S4 Effect of slurry flow rate on the FCDI desalination performance of CNFs@BiOCl-5. (a) Current response profiles of the FCDI system at different slurry flow rate. (b) Time-dependent variation of slurry flow rate in the desalination chamber. (c) Specific energy consumption at different slurry flow rate.
